# The mechanism of increased intestinal palmitic acid absorption and its impact on hepatic stellate cell activation in nonalcoholic steatohepatitis

**DOI:** 10.1038/s41598-021-92790-z

**Published:** 2021-06-28

**Authors:** Masakazu Hanayama, Yasunori Yamamoto, Hiroki Utsunomiya, Osamu Yoshida, Shuang Liu, Masaki Mogi, Bunzo Matsuura, Eiji Takeshita, Yoshiou Ikeda, Yoichi Hiasa

**Affiliations:** 1 Department of Gastroenterology and Metabology, Ehime University Graduate School of Medicine, 454 Shitsukawa, Toon-shi, Ehime, 791-0295 Japan; 2 Department of Pharmacology, Ehime University Graduate School of Medicine, 454 Shitsukawa, Toon-shi, Ehime, 791-0295 Japan

**Keywords:** Diseases, Gastroenterology, Pathogenesis

## Abstract

Dietary palmitic acid (PA) promotes liver fibrosis in patients with nonalcoholic steatohepatitis (NASH). Herein, we clarified the intestinal absorption kinetics of dietary PA and effect of trans-portal PA on the activation of hepatic stellate cells (HSCs) involved in liver fibrosis in NASH. Blood PA levels after meals were significantly increased in patients with NASH compared to those in the control. Expression of genes associated with fat absorption and chylomicron formation, such as CD36 and MTP, was significantly increased in the intestine of NASH model rats compared with that in the controls. Plasma levels of glucagon-like peptide-2, involved in the upregulation of CD36 expression, were elevated in NASH rats compared with those in the controls. Furthermore, portal PA levels after meals in NASH rats were significantly higher than those in control and nonalcoholic fatty liver rats. Moreover, PA injection into the portal vein to the liver in control rats increased the mRNA levels associated with the activation of HSCs. Increased intestinal absorption of diet-derived PA was observed in NASH. Thus, the rapid increase in PA levels via the portal vein to the liver may activate HSCs and affect the development of liver fibrosis in NASH.

## Introduction

Non-alcoholic fatty liver disease (NAFLD) is a chronic liver disease. About 20% of patients with NAFLD develop non-alcoholic steatohepatitis (NASH) with hepatitis and fibrosis^1^. Some patients with NASH are expected to have cirrhosis and hepatocellular carcinoma with a poor prognosis. The prevalence of NASH is increasing worldwide^2^.

Fatty acids (FAs) play an important role in the pathogenesis of NASH. Long-chain fatty acids (LCFAs) induce lipid deposition, inflammation, and production of reactive oxygen species in the liver^3,4^. In particular, palmitic acid (PA) is strongly lipotoxic for the liver^5,6^. Dietary FAs and intestinal chylomicrons (CMs) are among the major sources of PA in NASH^7,8^.

Previous studies have shown that the intestinal absorption of ^13^C-labeled palmitate was significantly higher in patients with NASH than in healthy controls, and a strong association with clinical parameters related to hepatic steatosis and fibrosis in the early stage of NASH has been reported^9^. In another study, increased absorption of dietary PA was observed in patients with cirrhosis^10^. Liver fibrosis is mediated by activated hepatic stellate cells (HSCs) during tissue repair^11^, and there are several reports on the effect of PA in the activation of HSCs^12,13^. However, the contribution of dietary PA to the pathogenesis of NASH, especially in the context of HSC activation, remains unknown. We hypothesized that the overflow of dietary PA to the liver affects the HSCs in NASH.

In this study, we investigated the mechanism underlying increased intestinal absorption of PA using a rat model of NASH and evaluated the effect of dietary PA administered via the portal vein on the activation of HSCs in NASH.

## Results

### Serum PA levels are increased in patients with NASH after meals

**Figure1:**
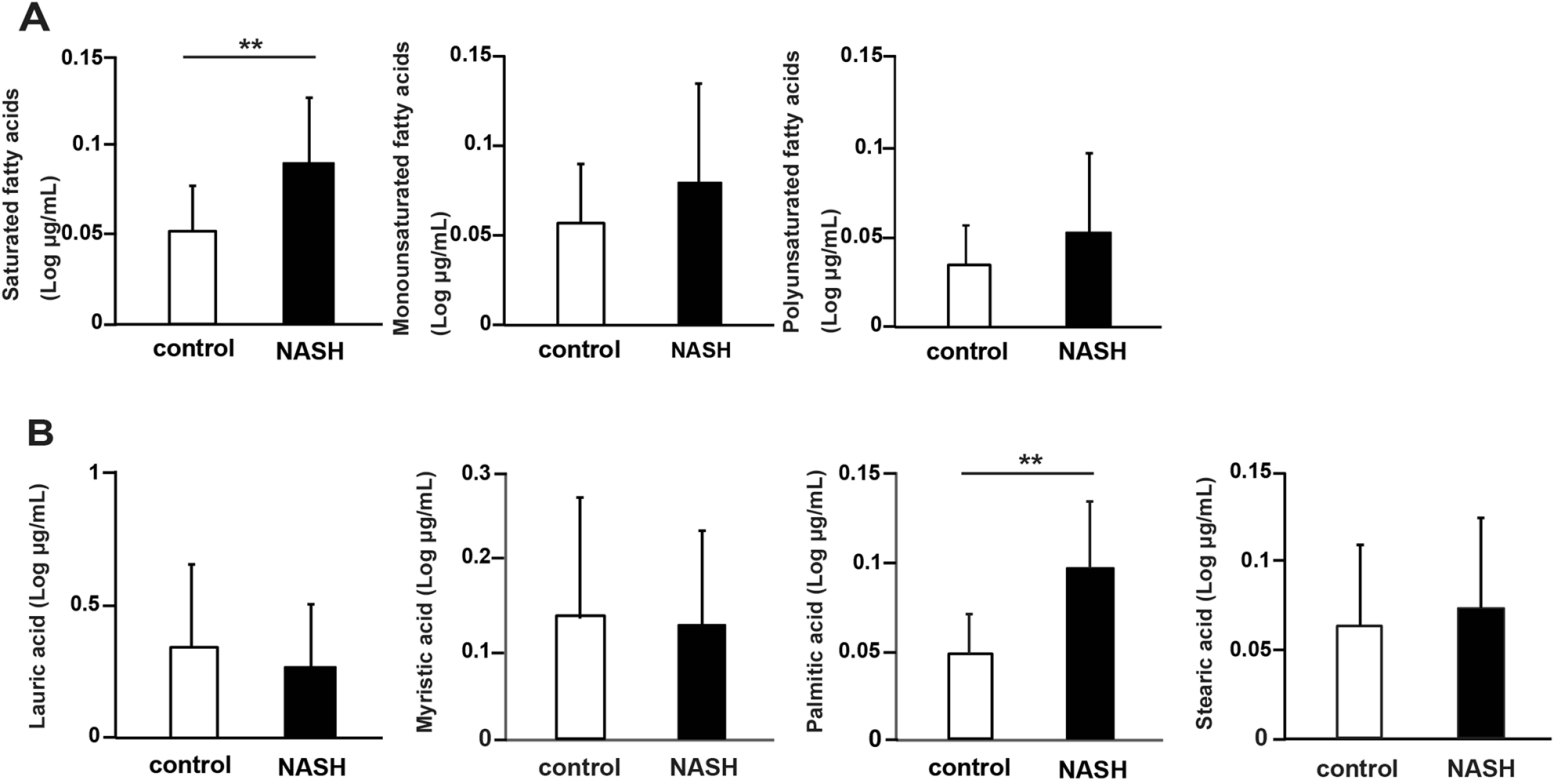
Relative change in fatty acid levels in human patients with nonalcoholic steatohepatitis (NASH; N = 41) and controls (N = 39). (**A**) Relative changes in saturated fatty acid (SFA), monounsaturated fatty acid, and polyunsaturated fatty acid levels. (**B**) Relative changes in lauric, myristic, palmitic, and stearic acid levels in the fraction with SFAs. Data in bar plots are shown as the mean ± standard deviation, and significant differences were determined using the Mann–Whitney U-test.**P < 0.01.

The patient background is shown in Supplemental Table S1. Patients without liver disease other than fatty liver, no gastrointestinal disorders, or history of heavy drinking were included. The exclusion criteria are shown in Supplementary Figure S1. First, we measured the change in FA levels in the serum before and after meals, which revealed that only saturated fatty acids (SFAs) were significantly increased in patients with NASH compared to those in controls (P < 0.01, Fig. 1A). Furthermore, measurement of the changes in each fraction of SFAs showed that among the SFAs, only PA levels were significantly increased (P < 0.01, Fig. 1B). Based on these results, we decided to focus on intestinal absorption of PA.

### NASH rats exhibit liver fibrosis and activation of HSCs

**Figure 2:**
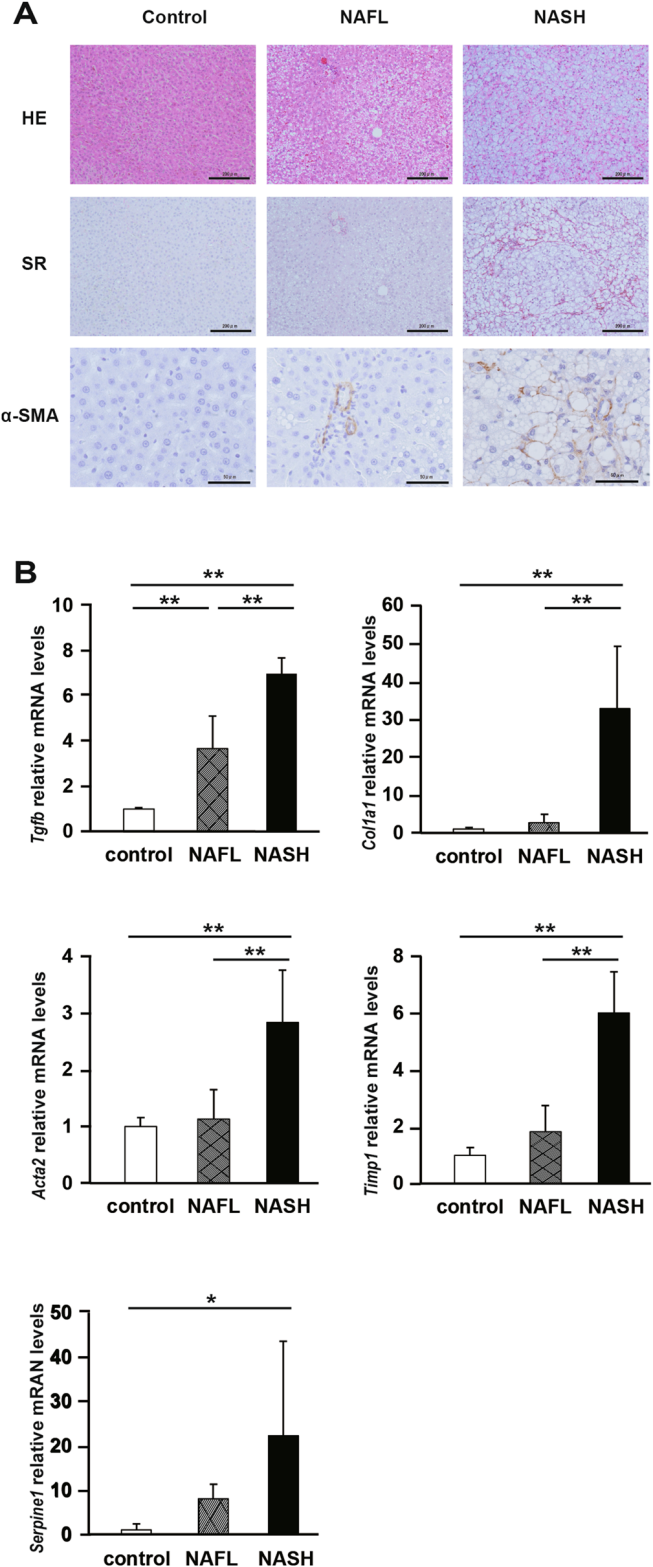
Liver fibrosis and activationoh hepatic stellate cells (HSCs) in nonalcoholic steatohepatitis (NASH) rats. (**A**) Representative images of liver sections stained with hematoxylin and eosin (H&E), Sirius Red stain, and anti-α-SMA antibody. (**B**) The mRNA levels of liver fibrosis markers, transforming growth factor-β (Tgfb), collagen 1a1 (Col1a1), α-smooth muscle actin (Acta2), tissue inhibitor of metalloproteinase 1 (Timp1), and plasminogen activator inhibitor 1 (Serpine1). N = 5 per group. Statistical analysis among the three groups was performed using Kruskal–Wallis ANOVA. Only when a significant difference was found, Tukey's multiple comparison test was performed. *P < 0.05, **P < 0.01.

We fed Sprague–Dawley (SD) normal rats a high-fat diet (HFD)/high-fat cholesterol diet (HFCD) to generate nonalcoholic fatty liver (NAFL)/NASH rat models. In subsequent experiments, we used SD, NAFL, NASH rats for validation. First, we performed hematoxylin and eosin (H&E) staining, Sirius Red staining, and immunostaining for α-smooth muscle actin (SMA) in liver tissues to evaluate fat deposition and liver fibrosis in each group. Thereafter, we performed RT-PCR analysis of genes related to the activation of HSCs. In tissue staining, fat deposits and α-SMA-positive cells were observed in the liver of NAFL and NASH rats (Fig. 2A, Supplementary Fig. S2). The degree of liver steatosis and fibrosis, lobular inflammation, and hepatocellular ballooning in each group is shown in Supplementary Table S2. Moreover, liver fibrosis was seen in NASH rats. These results show that HFD/HFCD fed rats exhibited characteristics of the NAFL/NASH model.

Next, we measured the mRNA levels of the markers of HSCs activation. In NAFL and NASH rats, the expression of *Tgfb* mRNA was significantly higher than that in control rats (P < 0.01). The expression of *Col1A1*, *Acta2*, *Timp1* mRNAs was significantly higher in NASH rats than that in NAFL and controls rats (P < 0.01). The expression of *Serpine1* mRNA was significantly higher in the NASH rats than that in control rats (*P* < 0.05) (Fig. 2B). These results show that HSCs were activated and remarkable fibrosis occurred in the liver of NASH rats.

### PA absorption increases in the intestine of NASH model rats

**Figure 3:**
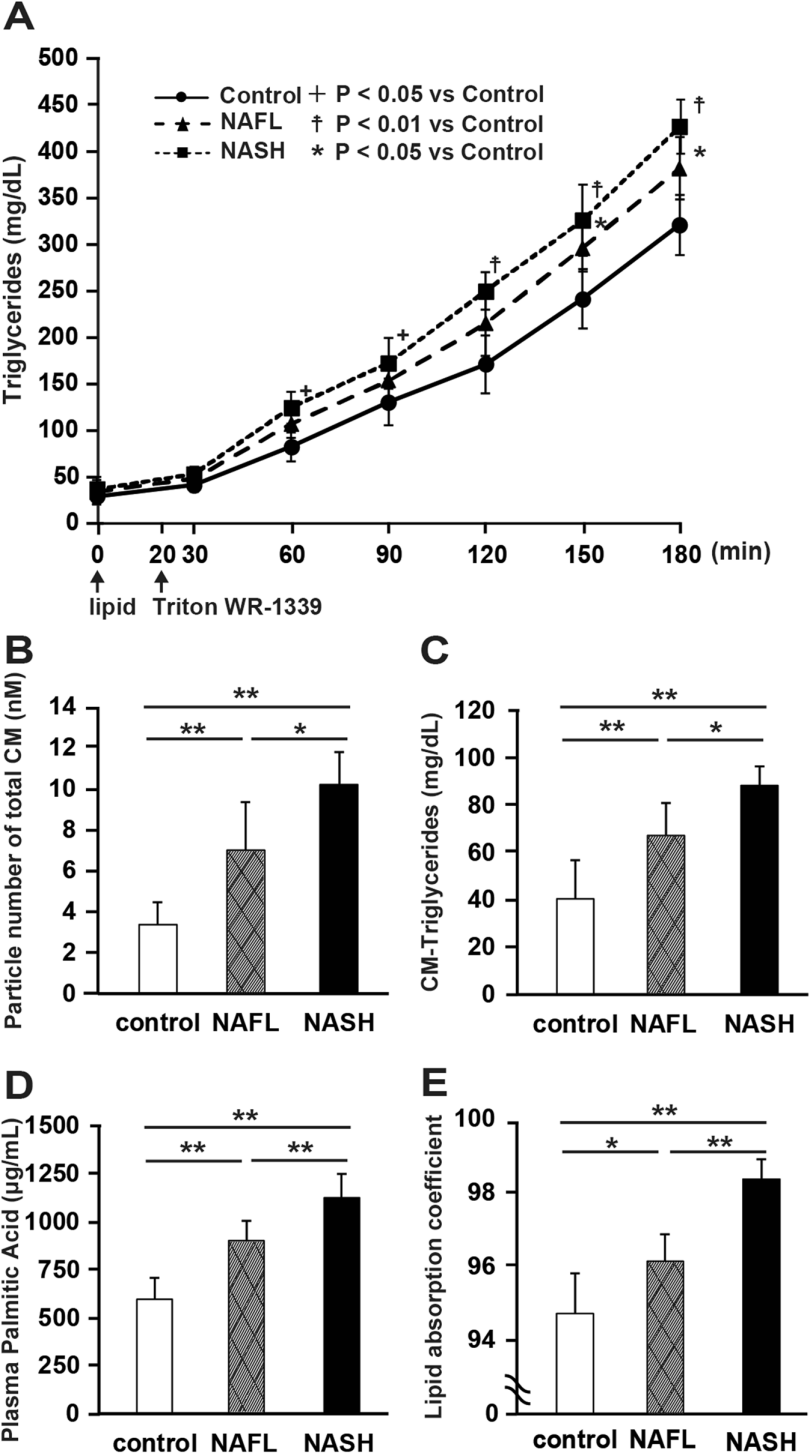
Increase in lipid and fatty acid absorption in the intestine of nonalcoholic steatohepatitis (NASH) rats. (**A**) Time-dependent increase in triglyceride (TG) level in the plasma of each model rat upon infusion of lipid emulsion and Triton (N = 6 per group; + P < 0.05, NASH vs. control; *P < 0.05, NAFL vs. control; ☨P < 0.01, NASH vs. control). Changes in (**B**) total chylomicron (CM) particle number, (**C**) chylomicron TG (CM-TG) concentration, (**D**) palmitic acid concentration before and 180 min after the meal using HPLC and gas chromatography, respectively (N = 6 per group; *P < 0.05; **P < 0.01). (**E**) Lipid absorption coefficient (LAC) in each group. LAC was measured when all the three groups of animals were fed the same diet (MFD) (N = 6 per group; *P < 0.05, **P < 0.01). Data in bar plots are shown as the mean ± standard deviation. Statistical analysis between the three groups was performed using Kruskal–Wallis ANOVA. Tukey's multiple comparison test was performed only when a significant difference was found.

We continuously administered a lipid emulsion to the duodenum of each model rat and monitored the concentration of triglycerides (TGs) in the portal plasma. We also administered Triton WR-1339 intravenously for blocking lipoprotein catabolism. After 180 min, the TG concentration in the portal plasma was significantly higher in NASH (419.2 ± 28.9 mg/dL; P < 0.01) and NAFL (370.8 ± 33.7 mg/dL; P < 0.05) rats than in control rats (317.9 ± 32.2 mg/dL) (Fig. 3A).

Next, we determined the total number of chylomicron (CM) particles and the concentration of TGs present in the plasma CM (CM-TG) using high-performance liquid chromatography (HPLC). The total number of CM particles (Fig. 3B) and CM-TG concentration (Fig. 3C) were significantly higher in NASH rats than those in NAFL and control rats (P < 0.01 and P < 0.05 compared with control and NAFL rats, respectively). The total number of CM particles and CM-TG concentration were also significantly higher in NAFL rats compared with that in control rats (P < 0.01). Similarly, the concentration of PA in the portal vein after administration of the lipid emulsion (Fig. 3D) was significantly higher in NASH rats than that in NAFL and control rats (P < 0.01, compared with control and NAFL rats). In addition, we measured the lipid absorption coefficient (LAC) in all the three groups of rats fed the same diet (MF normal died). The LAC was significantly higher in NASH rats than that in NAFL and control rats (P < 0.01, respectively) (Fig. 3E). The LAC was also significantly higher in NAFL rats than that in control rats (P < 0.05).

### Genes involved in the absorptionof FAs are highly expressed in the intestine of NASH rats

**Figure 4:**
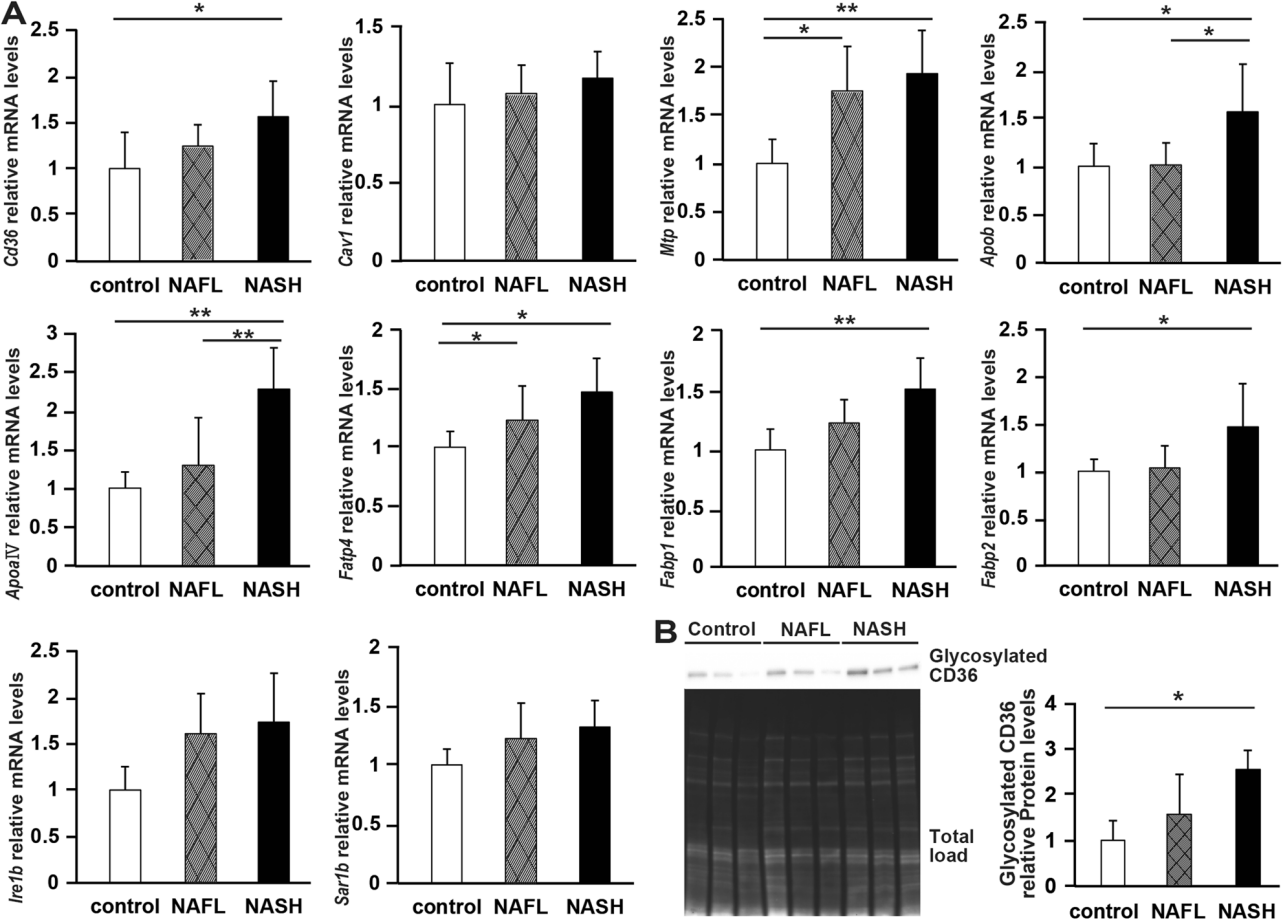
Expression of genes and proteins involved in fatty acid and lipid absorption in the intestine. (**A**) The mRNA levels of the following genes were related to the transport of long-chain fatty acids and lipids: cluster of differentiation (Cd36), caveolin 1 (Cav1), microsomal triglyceride transfer protein (Mtp), apolipoprotein B (Apob), apolipoprotein A-IV (Apoa-IV), fatty acid transporter protein 4 (Fatp4), fatty acid-binding protein (Fabp), including liver FABP (Fabp1) and intestinal FABP (Fabp2), inositol-requiring enzyme1b (Ire1b), and secretion associated, Ras-related GTPase1b (Sar1b). N = 6 per group. (**B**) Western blot analysis of glycosylated CD36 in jejunal tissue. N = 3 per group. Data in bar plots are shown as the mean ± standard deviation. Statistical analysis between the three groups was performed using Kruskal–Wallis ANOVA. Tukey's multiple comparison test was performed only when a significant difference was found. *P < 0.05, **P < 0.01.

We used jejunal tissue to evaluate the expression of genes that contribute to the absorption and synthesis of FAs and lipids via CM (Fig. 4A)^14–21^. The expression levels of *Cd36*, *Fabp1*, and *Fabp2* mRNAs, the latter two being involved in FA transport, were significantly higher in NASH rats than in control rats (P < 0.05, P < 0.01, and P < 0.05, respectively ). By contrast, the expression level of *Cav1* mRNA was not significantly different among the three groups. The expression levels of *Mtp* mRNA involved in the formation of CMs, as well as those of *Fatp4* mRNA, were significantly higher in NASH and NAFL rats than in control rats (P < 0.01 and P < 0.05, respectively), whereas *Apoa-IV* and *Apob* mRNA levels were significantly higher in NASH rats than in the other two groups of rats (P < 0.01 and P < 0.05, respectively). The mRNA expression levels of *Ire1b* and *Sar1b*, which are involved in the formation of CMs, did not vary significantly among these groups of rats (Fig. 4A). Next, we then analyzed the level of glycosylated CD36 in jejunal tissues (Fig. 4B, Supplementary Fig. S3) and found that the expression of glycosylated CD36, an activated form of CD36, was significantly higher in NASH rats than in control rats.

### Glucagon-like peptide-2 (GLP-2) promotes intestinal lipid absorption

**Figure 5:**
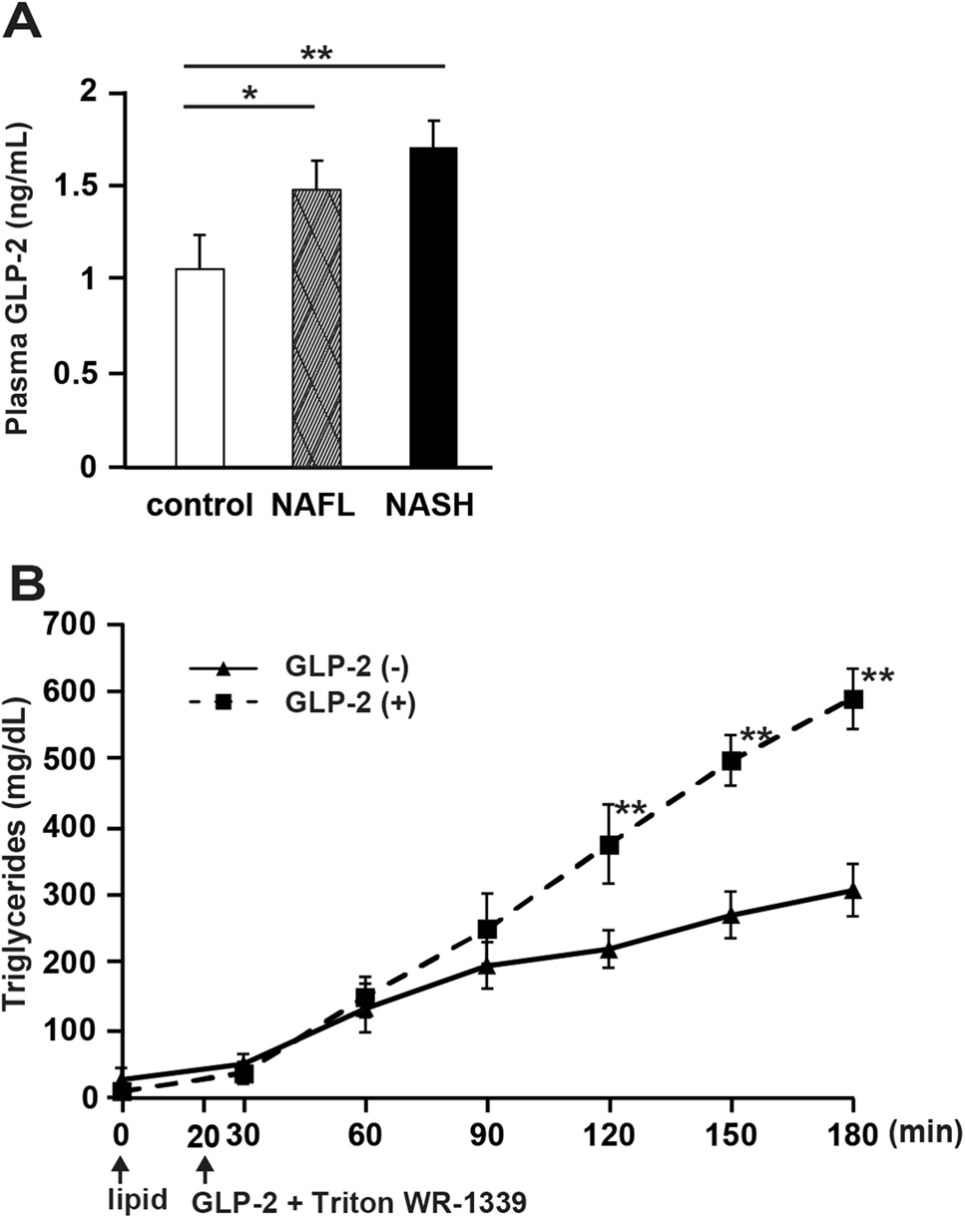
Promotion of intestinal lipid absorption by glucagon-like peptide-2 (GLP-2). (**A**) Fasting blood GLP-2 levels in each group (N = 5 per group;). (**B**) Time-dependent increase in plasma triglyceride (TG) level in each model rat administered lipid emulsion and Triton infusion, as required. GLP-2 was administered 20 min after the administration of the lipid emulsion. N = 6 per group. Data in bar plots are shown as the mean ± standard deviation. Significant differences were indicated by Student's t-test. *P < 0.05, **P < 0.01.

The plasma level of GLP-2, which activates intestinal CD36 by glycosylation and increases the absorption of lipids in the intestine^22^, was measured at the time of fasting in all the model rats. The plasma concentration of GLP-2 was significantly higher in NASH (P < 0.01) and NAFL rats (P < 0.05) than in control rats (Fig. 5A). Next, we administered GLP-2 intraperitoneally to normal SD rats and evaluated the concentration of TGs in the portal vein. The TG concentration in the portal vein was significantly increased in the GLP-2 administration group (P < 0.01; Fig. 5B).

### An increase in trans-portal PA activates HSCs

**Figure 6:**
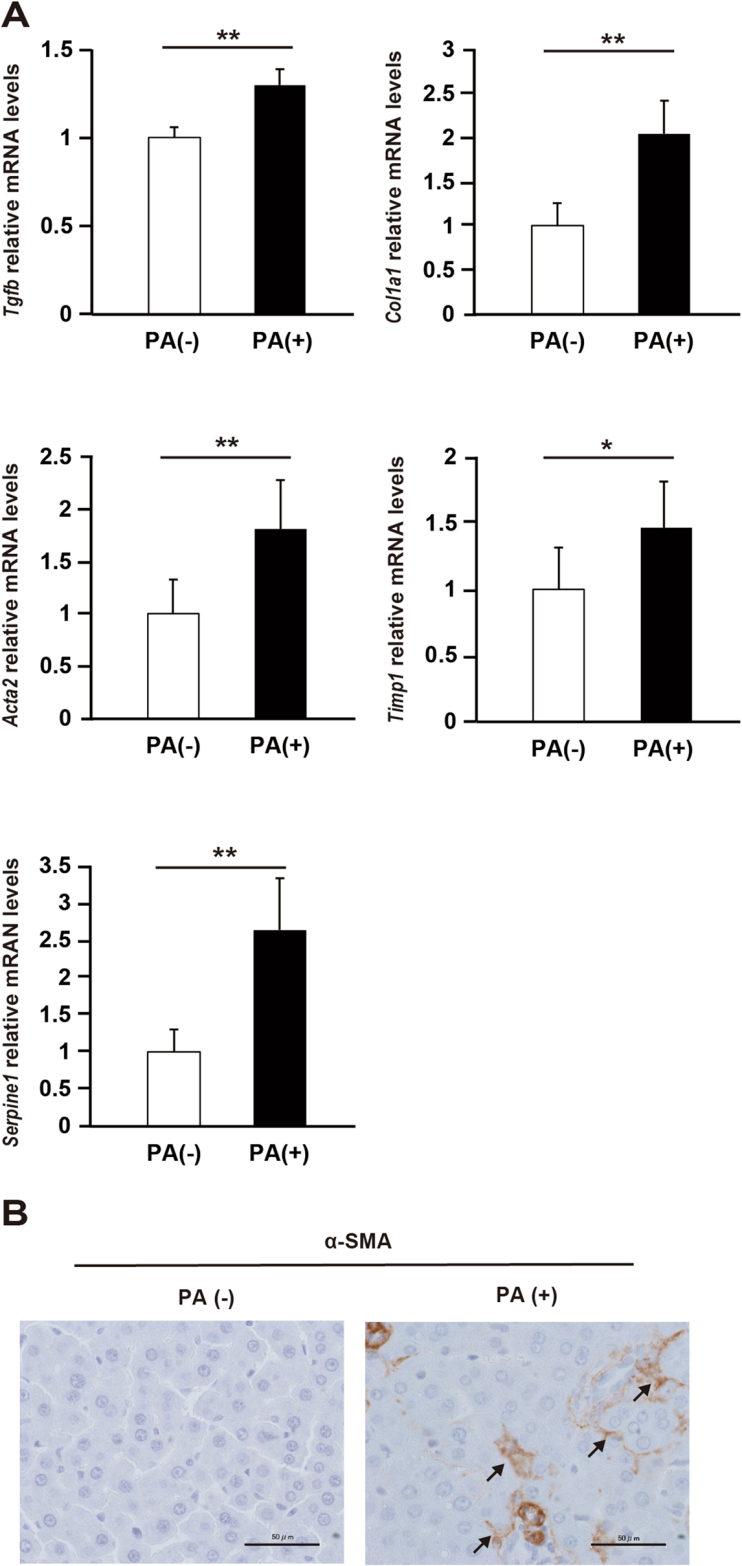
Activation of hepatic stellate cells (HSCs) by palmitic acid (PA). (**A**) The mRNA levels of liver fibrosis markers, transforming growth factor-β (Tgfb), collagen 1a1 (Col1a1), α-smooth muscle actin (Acta2), tissue inhibitor of metalloproteinase 1 (Timp1), and plasminogen activator inhibitor 1 (Serpine1). N = 6 per group; *P < 0.05. **P < 0.01. Data in bar plots are expressed as the mean ± standard deviation. Significant differences were indicated by Mann–Whitney U-test. (**B**) Immunohistochemistry of α-SMA expression in rat liver using anti-α-SMA antibody. PA( +), PA solution group; PA(–), PA vehicle solution group.

We injected PA into the liver of normal SD rats via the portal vein and assessed the expression of genes related to HSC activation (Fig. 6A). Administration of PA significantly increased the levels of *Col1A1, Acta2, Tgfb, Timp*, and *Serpine1* mRNAs in the liver (*P* < 0.01). Furthermore, PA administration increased the expression of α-SMA in the liver (Fig. 6B, Supplementary Fig. S4).

## Discussion

This study was designed to evaluate the kinetics of PA absorption in the intestine and to evaluate the effect of PA, administered via the portal vein, on the activation of HSCs in pathogenesis of NASH. We found that the blood PA levels after meals were significantly increased in patients with NASH compared with that in controls (P < 0.01). Similarly, the concentration of PA after meal in the portal vein in NASH model rats was increased. The increase in PA was caused by the upregulation of molecules related to the absorption of PA in the intestine. Furthermore, we demonstrated that the influx of PA to the liver activated HSCs involved in liver fibrosis. To the best ou our knowledge,this is the first study to report that increased intestinal absorption of PA affects liver fibrosis in NASH.

Lipotoxicity and the gut-liver axis play an important role in the development and progression of NASH^23^. PA, a saturated FA, is a major component of dietary FA and has potent toxic effects^24^. It has been previously reported that fasting blood PA levels are increased in patients with NASH^25^. In the present study, we confirmed the increase in blood PA levels in patients with NASH not only during fasting but also after meals. These results suggest that FAs in the diet may have a great influence on the pathogenesis of NASH. Therefore, we focused on the intestinal absorption of dietary PA. We found that the portal PA levels were higher in the NASH group than in the control and NAFL groups (P < 0.01, compared with control and NAFL rats). Dietary LCFA, including PA, are converted into TGs at the endplasmic reticulum of intestinal cells and are incorporated into CMs^26,27^. Thus, we measured the number of CM particles and the CM-TG concentration in the blood after meals, and found that the number of CM particles and CM-TG concentrations were higher in NASH rats than in control and NAFL rats (P < 0.01 and P < 0.05 compared with control and NAFL rats, respectively). These results suggest that increased absorption of dietary PA in the intestine resulted in elevated blood PA levels in NASH patients.

Next, we examined the mechanism of increased lipid and FA absorption and increased CM formation in the rat jejunum. The expression of molecules related to lipid absorption and CM synthesis was significantly higher in NASH model rats than in the control and NAFL rats. We found that the levels of both CD36 mRNA and glycosylated protein in the intestine were elevated. Highly glycosylated CD36 is expressed in the apical membrane of enterocytes^22^, and CD36 has been implicated in the absorption of LCFA, as well as in the formation and secretion of CM in the intestine^27–32^. We have examined the expression of factors associated with the absorption of FAs in the jejunum of NASH rats; however, we have no data regarding the increased compromised intestinal barrier in this model. Our data suggest that CD36, upregulated in the intestine of NASH rats, contributes to the increased absorption of PA in the intestine.

GLP-2 is known to increase the intestinal absorption of FAs by increasing the expression of CD36 and by promoting its glycosylation^22^. GLP-2 also increases the absorption of FAs via the formation of ApoB48 lipoprotein and CMs^22,33^. In fact, continuous administration of GLP-2 enhances [3H]triolein uptake in the circulating blood from the intestine^33^. Herein, we observed that plasma GLP-2 levels were elevated in NASH model rats. In fact, when GLP-2 analogs were administered to control rats, postprandial TG levels were found to be increased. Therefore, we speculated that GLP-2 regulates the absorption of LCFAs by controlling the intestinal expression of CD36 in NASH.

MTP is the rate-limiting enzyme in the synthesis of CMs, and CM-mediated FA secretion is highly dependent on MTP^30^. APOA-IV is involved in the uptake of TGs into the CMs^34^ and upregulates the expression of MTP at the mRNA and protein levels^35^. APOA-IV in the intestine is upregulated by the absorption of dietary LCFAs^31,36^. In our study, the expression of MTP and APOA-IV was significantly increased in the jejunum of NASH rats. These results suggest that the increased absorption of LCFA may induce higher expression of MTP via overexpression of APOA-IV in the NASH model rats.

PA directly activates macrophages via toll-like receptors and nuclear factor-κB, and activated macrophages exacerbate inflammation by releasing proinflammatory cytokines^37^. In the present study, postprandial blood PA levels were elevated in NASH rats, as well as in NAFL rats, compared with those in control rats. This result suggests that dietary PA triggers liver inflammation in the early stages of NAFLD. Our results also show that administration of PA via the portal vein to the liver increased the mRNA levels of molecules involved in the activation of HSCs. Absorbed LCFAs in the intestine generally flow into the liver via the portal vein as a component of CM^38^. PA has been reported to activate HSCs via the inflammasome and hedgehog signaling^12,13^. Activated HSCs are known to enhance the expression of TGF-β, COL1A1, α-SMA, TIMP, and PAI-1, which are involved in liver fibrosis^39–44^. Previous studies have shown that blood PA concentrations in patients with cirrhosis are associated with the degree of liver fibrosis and that PA concentrations are high even before the onset of cirrhosis^45,46^. In this study, we showed that intestinal absorption of PA and PA concentration in the portal blood were increased in NASH rats. Our results suggest that the influx of PA into the liver may activate HSCs and affect the progression of liver fibrosis in the late stages of NAFLD.

Certain limitations were noted in the current study. First, although we demonstrated that the increased intestinal absorption of PA caused hepatic activation of HSCs and liver fibrosis in NASH rats, we could not perform the same experiments in humans owing to the difficulty in obtaining human portal blood in a time-course manner. Second, we could not show the precise strategy for treating NASH by targeting lipid absorption; however, we are currently assessing the influence of genetic or pharmacological alteration of CD36 and MTP expression in rats on the absorption of PA and development of NASH.

**Figure 7:**
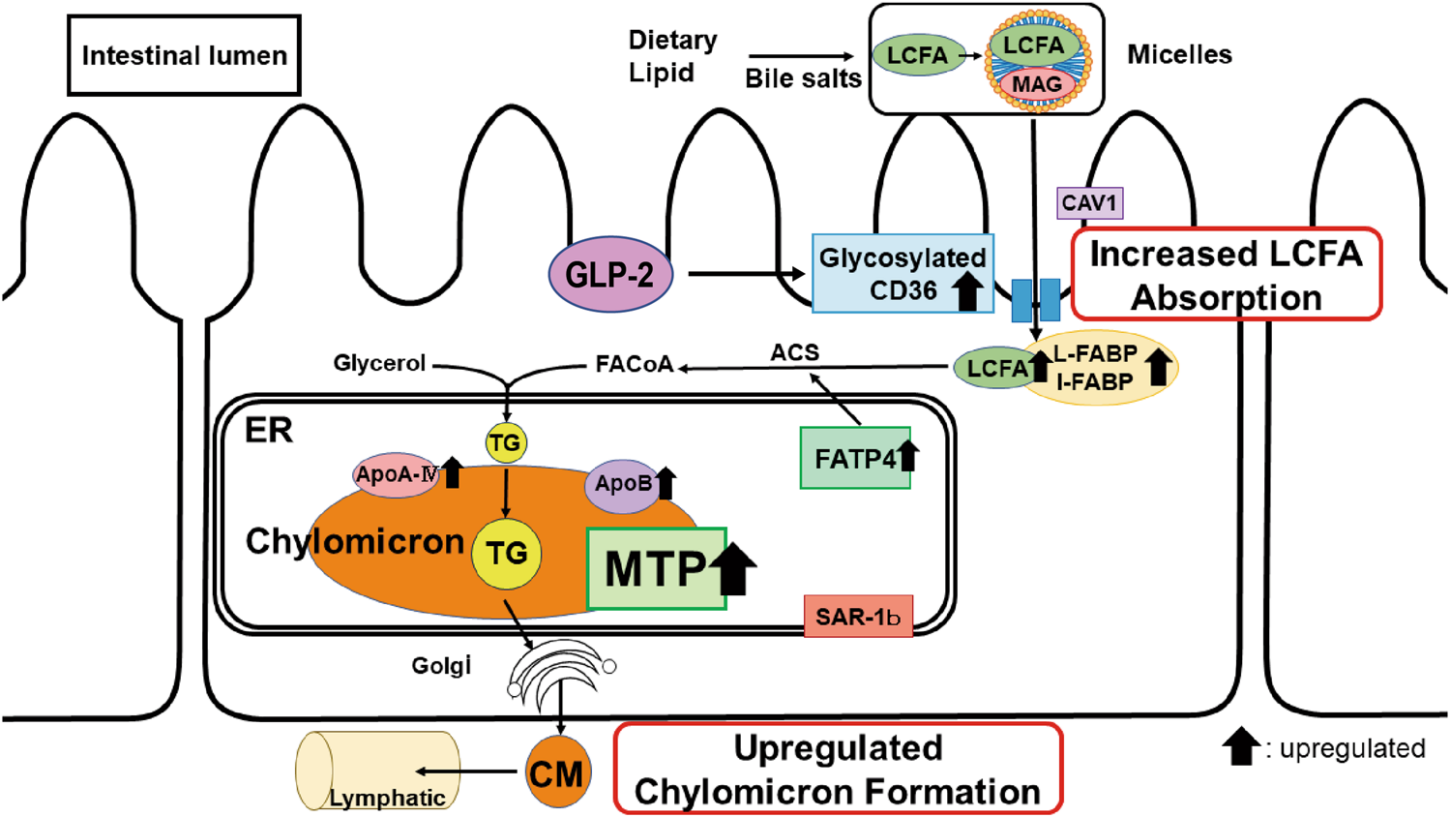
Model of lipid and fatty acid (FA) absorption in nonalcoholic steatohepatitis (NASH). First, the expression of CD36 was enhanced by glucagon-like peptide-2 (GLP-2) in the intestinal villi, followed by an increase in FA uptake into the intestinal cells. Second, triglycerides (TGs) were synthesized from long-chain fatty acids, which were incorporated into chylomicrons in the endoplasmic reticulum (ER) via microsomal triglyceride transfer protein (MTP). Third, the synthesized chylomicron was secreted from the ER and flowed into the lymphatic vessels via the Golgi apparatus. LCFA, Long-chain fatty acid; ACS, acyl-CoA synthetase; FA-CoA, fatty acyl-CoA.

In conclusion, absorption of PA increases in the jejunum of NASH rats owing to the upregulation of intestinal glycosylated CD36 and MTP by GLP-2 (Fig. 7). Furthermore, increased PA influx into the liver activates HSCs and promotes the development of liver fibrosis. Modulating intestinal absorption of PA by regulating CD36, MTP, and GLP-2 expression may reduce liver inflammation and inhibit the progression of liver fibrosis in NASH. Furthermore, this effect is expected to maintain the hepatic functional reserve and improve the prognosis of patients with NASH.

## Methods

### Human subjects

Experimental procedures were performed in accordance with the approved guidelines. The inclusion and exclusion criteria are shown in Supplementary Figure S4. A total of 182 subjects were recruited prospectively to the study at Ehime University Hospital (Ehime, Japan) between January 2012 and August 2015. Among 113 subjects diagnosed with fatty liver after ultrasonography during hospitalization for liver scrutiny, 85 subjects met the inclusion criteria, including the absence of gastrointestinal or other disorders, and were enrolled as patients with NAFLD. Three patients with NAFLD did not consent to liver biopsy. All biopsy samples were evaluated by a liver pathologist. NASH was confirmed according to Matteoni's classification, and 65 patients with Matteoni's types 3 and 4 were diagnosed with NASH^47^. After we explained the methods and objectives of the study, 24 of the patients with NASH refused to participate, leaving a total of 41 NASH patients. Among the 69 subjects who were not diagnosed with fatty liver using ultrasonography during hospitalization for health checkup, 49 subjects met the inclusion criteria. Thirty-nine subjects, excluding ten subjects who refused to participate in the study, were enrolled as healthy subjects.

The study was performed following a protocol approved by the Institutional Review Board of Ehime University Hospital (approval number: 1407006, University Hospital Medical Information Network ID: UMIN000014792). All participants in the study provided written informed consent, and the protocol of the study followed the guidelines of the Declaration of Helsinki.

### FA analysis

All subjects received nutritional management under inpatient care. Each subject consumed a test meal with a total energy content of 15 kcal/kg. The test meal provided approximately 25%, 15%, and 60% of energy from fat, protein, and carbohydrate, respectively. The test meals were hospital diets strictly controlled by the Department of Nutrition, Ehime University. Blood samples were taken from individuals during fasting and 2 h after the meal. Serum was collected from the blood, and serum FA concentrations were measured using gas chromatography. Gas chromatography measurements were performed at Shikoku Chuken (Shikoku Chuken, Kagawa, Japan). Briefly, serum lipids were separated using Folch's method^48^, and FAs were methylated with boron trifluoride and methanol. The methylated FAs were then analyzed using a capillary gas chromatograph (GC-17A; Shimadzu, Kyoto, Japan).

The change in FA concentration was calculated according to Eq. (1):

1 $${\text{Change}}\;{\text{in}}\;{\text{FA}}\;{\text{concentration}} = {\log}\left( {{\text{FA}}\;{\text{concentration}}\;{\text{after}}\;{\text{meal}} - {\text{fasting}}\;{\text{FA}}\;{\text{concentration}}} \right)$$

### Animals

All procedures were approved by the Ehime University Animal Care Committee. All studies were performed in compliance with the ARRIVE guidelines. Approximately 8-week-old male SD rats (CLEA Japan, Tokyo, Japan) were maintained undeer a 12 h light/dark cycle. The disease model rats were bred according to published methods^49^. Rats were randomly assigned to three groups and fed the assigned diets for 18 weeks as follows: (1) the control group was fed MF normal diet (MF; Oriental Yeast, Tokyo, Japan), (2) the NAFL group was fed a HFD (68% MF, 30% palm oil, 2% cholic acid), and (3) the NASH group was fed a HFCD (68% MF, 27.5% palm oil, 2.5% cholesterol, 2% cholic acid). Animals were provides with ad libitum access to water.

### Tissue sample preparation

Rats were fasted overnight. and then sacrificed under deep anesthetic control. Subsequently, liver and jejunum tissues were harvested and stored as follows: (1) one section was fixed in 10% formalin for 24 h and embedded in paraffin; (2) another section was immersed in RNA-later (Life Technologies, Carlsbad, CA, USA) overnight and stored at −20 °C until use; (3) the remaining tissue was quickly frozen using liquid nitrogen and stored at −80 °C.

### Determination of lipid and FA absorption and chylomicron production

All rats were made to fasted overnight before the procedure. Cannulas were placed in the portal vein and duodenum under isoflurane anesthesia. The rats were then infused with the lipid emulsion into the duodenum at a rate of 3 mL/h. The emulsion consisted of 12.5 μmol glycerol tripalmitate (Wako, Osaka, Japan), 57 μmol sodium taurocholate, and 7.8 μmol phosphatidylcholine in 3 mL of PBS^50^. Triton WR-1339 (0.5 g/kg) (Tyloxapol; Sigma–Aldrich Co, St. Louis, MO, USA) was administered 20 min after beginning the infusion of the emulsion. Blood was collected from the portal vein every 30 min for 3 h. Plasma triglyceride (TG) levels were measured using enzymatic kits (Triglyceride E-test; Wako, Osaka, Japan). Plasma CM levels were measured using high-performance liquid chromatography (HPLC) (LipoSEARCH; Skylight Biotech, Akita, Japan). Plasma PA levels were measured using gas chromatography.

### Determination of coefficient of intestinal lipid absorption

Twenty-four-hour stools were collected, and the twenty-four-hour intake of meals was measured. During the period when stool was collected, all three groups of rats were fed the same MF normal diet. Lipids in stool and feed were extracted using the Bligh and Dyer method^51^, and their respective lipid content was determined. Briefly, chloroform and methanol were added to the specimens in a ratio of 1:2, vortexed, and separated into two layers by centrifugation. The separated lower layer was collected, and then chloroform was dried to extract the lipids. The LAC was calculated according to Eq. (2):

2 $$LAC = ~\frac{{\left( {dietary~lipid~intake~ - lipid~defecation} \right)}}{{dietary~lipid~intake}}~ \times 100$$

### FA absorption after administration of glucagon-like peptide-2 (GLP-2)

After overnight fasting, blood was collected from the tail vein of rats in each group and the plasma was separated. Plasma GLP-2 concentration was measured using a rat GLP-2 ELISA kit (Yanaihara Institute, Fujinomiya, Japan). After overnight fasting, control rats were infused with the lipid emulsion at a rate of 3 mL/h into the duodenum, and Triton WR-1339 (0.5 g/kg) with or without the GLP-2 analog (0.25 mg/kg) (Bachem, Bubendorf, Switzerland) was administered after 20 min. Blood was collected from the portal vein every 30 min for 3 h and plasma TG levels were measured.

### Measurement of liver fibrosis markers after portal administration of PA

PA (0.0256 μg) was added to 1,000 μL 99% ethanol to prepare solution (A). Next, 1.0 g bovine serum albumin was mixed with 9 mL water and 40 μL 1 N NaOH solution (B). Thereafter, 100 μL solution (A) was added to 900 μL solution (B) to prepare the PA solution. PA solution or PA vehicle solution were administered under anesthesia into the portal vein of control rats, and wounds were sutured after the procedure was completed. The rats were euthanized 24 h after the administration, and the liver tissue was collected. Expression of liver fibrosis markers was determined using real-time PCR.

### Real-time PCR

RNA was extracted from the jejunum and liver using a RNeasy Plus mini kit (Qiagen, Hilden, Germany). Reverse transcription reactions were performed using the High Capacity cDNA reverse transcription kit (Thermo Fisher Scientific, Waltham, MA, USA) according to the manufacturer’s instructions. Real-time PCR was performed using the LightCycler 480II (Roche Diagnostics, Mannheim, Germany). The annealing temperatures of the primers used are provided in Supplementary Table S3. Gene expression data were normalized to the expression of the $\mathrm{\beta }$-actin gene and expressed as a ratio of the values obtained for control rats.

### Western blot analysis

Rat jejunum tissue was homogenized using a TissueLyser (Qiagen) with modified radioimmunoprecipitation assay (RIPA) buffer (10 mmol/L Tris–HCl, pH7.4, 1% SDS, 0.5% NP40, and 150 mmol/L NaCl). Protein content was analyzed using the Lowry assay. Equal amounts of protein extracts were mixed with sample buffer (69.45 mmol/L Tris–HCl, pH6.8, 11.1% (v/v) glycerol, 1.1% LDS, 2.5% 2-mercaptoethanol, 0.005% bromophenol blue) at 95 °C for 5 min. Samples were electrophoresed on Mini-PROTEAN TGX 7.5% stain-free gels (Bio-Rad, California, USA) and the proteins were blotted onto Immuno-Blot low fluorescence polyvinylidene fluoride (PVDF) membranes. Stain-free technology (Bio-Rad) was used to determine equivalent loading. Membranes were blocked using 1.25% non-fat dry milk for 1 h at room temperature. After overnight exposure to the primary antibody overnight at 4 °C, the membrane was incubated with horse radish peroxidase (HRP)-conjugated secondary antibody and visualized using the ChemiDoc Touch imaging system (Bio-Rad). Quantification was performed using the ImageLab 5.0 software (Bio-Rad). Bands intensity was normalized to the concentration of total protein as previously reported^52^. The following antibodies were used to detect CD36: anti-CD36 (LSBio #LS-B662) and anti-rabbit HRP-conjugated IgG (GE Healthcare #NA934).

### Tissue histology and immunohistochemical staining

Formalin-fixed liver tissues were stained with H&E. The tissue was then stained with Sirius Red in picric acid to assess liver fibrosis. Liver tissues were incubated for 1 h at room temperature with an aqueous solution of saturated picric acid containing hematoxylin^53^.

Formalin-fixed, paraffin-embedded rat liver tissues were used for immunostaining. Immunostaining was performed using polyclonal α-SMA (1:200; Thermo Scientific # RB-9010) as the primary antibody; MAX-PO(R) (Nichirei #414,181) was used as the secondary antibody. After immunostaining, the colors was developed using 3,3ʹ-diaminobenzidine (DAB) chromogen, and the tissue sections were examined. The density of α-SMA was determined by counting the total number of α-SMA positive cells per field (5 sections per animal, N = 5 animals/group).

### Statistical analysis

Results are presented as mean ± standard deviation. In the analysis between two groups, normally distributed and skewed data were analyzed using Student's t-test and Mann–Whitney U-test, respectively. The three groups were statistically compared using Kruskal–Wallis ANOVA. Only when a significant difference was found, Tukey's multiple comparison test was performed. All statistical analyses were performed using R (The R Foundation for Statistical Computing, Vienna, Austria).

## Supplementary Information

Supplementary Information.

## Data Availability

The datasets generated during and/or analyzed during the current study are available from the corresponding author on reasonable request.
