# Supplementary material for: The mechanism of increased intestinal palmitic acid absorption and its impact on hepatic stellate cell activation in nonalcoholic steatohepatitis
Source: Sci Rep. 2021 Jun 28;11:13380. doi: 10.1038/s41598-021-92790-z (PMC8239050; doi:10.1038/s41598-021-92790-z)
Supplement: Supplementary file 1 — Supplementary Information. [file 41598_2021_92790_MOESM1_ESM.docx]

**Supplementary Information**

**The mechanism of Increased Intestinal Palmitic Acid Absorption and the impact on Hepatic Stellate Cell Activation in Non-alcoholic Steatohepatitis**

Masakazu Hanayama, Yasunori Yamamoto, Hiroki Utsunomiya, Osamu Yoshida, Shuang Liu, Masaki Mogi, Bunzo Matsuura, Eiji Takeshita, Yoshiou Ikeda, Yoichi Hiasa*

**Supplemental Table S1.** Clinical characteristics of the study subjects

| Clinical characteristics | Control subjects (n=39) | NASH patients (n=41) | P value |
| --- | --- | --- | --- |
| Age (years) | 68 ± 10 | 63 ± 12 |  |
| Sex (male : female) | 24 : 15 | 19 : 22 |  |
| Body mass index (kg/m^2^) | 22.9 ± 2.7 | 28.4 ± 4.9 | P < 0.01 |
| Platelet (10^4^/lL) | 19.9 ± 6.9 | 17.5 ± 7.2 | P < 0.05 |
| PT (%) | 97.4 ± 15 | 93.9 ± 19.6 |  |
| Total bilirubin (mg/dL) | 0.7 ± 0.3 | 0.8 ± 0.2 | P < 0.01 |
| Albumin (mg/dL) | 4.0 ± 0.6 | 4.0 ± 0.7 |  |
| AST (IU/L) | 21 ± 9 | 51 ± 40 | P < 0.01 |
| ALT (IU/L) | 18 ± 9 | 51 ± 42 | P < 0.01 |
| ALP (IU/L) | 231 ± 80 | 270 ± 132 |  |
| γ-GTP (IU/L) | 25± 17 | 64 ± 50 | P < 0.01 |
| Total cholesterol (mg/dL) | 178 ± 34 | 185 ± 39 |  |
| LDL cholesterol (mg/dL) | 105± 29 | 116 ± 34 |  |
| HDL cholesterol (mg/dL) | 42 ± 19 | 47 ± 13 |  |
| Triglyceride (mg/dL) | 80 ± 51 | 123 ± 64 | P < 0.01 |
| Type IV collagen 7s (ng/mL) | 4.1 ± 1.6 | 6.0 ± 2.5 | P < 0.01 |
| M2BPGi (cutoff index) |  | 1.6 ± 2.2 |  |
| Fibrosis (n) |  |  |  |
| 1 |  | 15 |  |
| 2 |  | 4 |  |
| 3 |  | 9 |  |
| 4 |  | 13 |  |
| Steatosis (n) |  |  |  |
| 0 |  | 1 |  |
| 1 |  | 13 |  |
| 2 |  | 16 |  |
| 3 |  | 11 |  |
| Lobular inflammation (n) |  |  |  |
| 0 |  | 3 |  |
| 1 |  | 18 |  |
| 2 |  | 16 |  |
| 3 |  | 4 |  |
| Hepatocellular ballooning (n) |  |  |  |
| 0 |  | 6 |  |
| 1 |  | 21 |  |
| 2 |  | 14 |  |

Significant differences were indicated by Mann-Whitney U test.

Data in table are presented as the mean ± standard deviation (SD) or as the number of subjects, as appropriate.

M2BPGi, Mac-2-binding protein glycosylation isomer,

Fibrosis, steatosis, lobular inflammation and hepatocellular ballooning were assessed according to Matteoni's classification.

**Supplemental Table S2.** Histological findings in the liver of rats

|  | Control (n = 6) | NAFL (n = 6) | NASH (n = 6) |
| --- | --- | --- | --- |
| Fibrosis (n) |  |  |  |
| 0 | 6 | 6 | 0 |
| 1 | 0 | 0 | 0 |
| 2 | 0 | 0 | 1 |
| 3 | 0 | 0 | 2 |
| 4 | 0 | 0 | 3 |
| Steatosis (n) |  |  |  |
| 0 | 6 | 0 | 0 |
| 1 | 0 | 5 | 0 |
| 2 | 0 | 1 | 0 |
| 3 | 0 | 0 | 6 |
| Lobular inflammation (n) |  |  |  |
| 0 | 6 | 4 | 3 |
| 1 | 0 | 2 | 2 |
| 2 | 0 | 0 | 1 |
| 3 | 0 | 0 | 0 |
| Hepatocellular ballooning (n) |  |  |  |
| 0 | 6 | 5 | 0 |
| 1 | 0 | 1 | 0 |
| 2 | 0 | 0 | 6 |
|  |  |  |  |

Fibrosis, steatosis, lobular inflammation and hepatocellular ballooning were assessed according to Matteoni's classification.

**Supplemental Table S3.** Primers used for real-time RT-PCR

| Target mRNA | Primer |  | Sequence (5'-3') | Annealing Temperature (℃) |
| --- | --- | --- | --- | --- |
| Transforming growth factor beta  (TGF-β) | *Tgfb* | forward | CCTGGAAAGGGCTCAACAC | 60 |
|  |  | reverse | TGCCGTACACAGCAGTTCTT |  |
| Collagen1a1 (Col1a1) | *Col1a1* | forward | GTGGACAGGCTGGTGTGAT | 60 |
|  |  | reverse | GGGACACCTCGTTCTCCAG |  |
| Alpha smooth muscle actin (α-SMA) | *Acta2* | forward | GCTCCGGGCTCTGTAAGG | 60 |
|  |  | reverse | GCCCATTCCAACCATCACT |  |
| Tissue inhibitor of metalloproteinase 1 (TIMP1) | *Timp1* | forward | TGCAACTCGGACCTGGTTAT | 60 |
|  |  | reverse | AGCGTCGAATCCTTTGAGCA |  |

| Target mRNA | Primer |  | Sequence (5'-3') | Annealing Temperature (℃) |
| --- | --- | --- | --- | --- |
| Plasminogen activator inhibitor 1  (PAI-1) | *Serpine1* | forward | AGAGCCAATCACAAGGCACT | 60 |
|  |  | reverse | GAGGCAAGTGAGGGCTGA |  |
| CD36 | *Cd36* | forward | GCGACATGATTAATGGCACA | 60 |
|  |  | reverse | TGGACCTGCAAATGTCAGAG |  |
| Caveolin1 (CAV1) | *Cav1* | forward | GGCAGACGAGGTGAATGAGA | 60 |
|  |  | reverse | TCCAGATGCCGTCGAAACTG |  |
| Microsomal triglyceride transfer protein (MTP) | *Mtp* | forward | GCGAGTCTAAAACCCGAGTG | 60 |
|  |  | reverse | CACTGTGATGTCGCTGGTTATT |  |
| Apolipoprotein B (Apo-B) | *Apob* | forward | CCCATCAGCACAAGTGTCAG | 60 |
|  |  | reverse | GCTGCTGATTAGAGTTGACAAGG |  |

| Target mRNA | Primer |  | Sequence (5'-3') | Annealing Temperature (℃) |
| --- | --- | --- | --- | --- |
| Apolipoprotein A-IV (APOA-IV ) | *ApoaIV* | forward | ACCCAGCTAAGCAACAATGC | 60 |
|  |  | reverse | AAGTTTGTCCTGGAAGAGGGTA |  |
| Fatty acid transport protein 4 (FATP4) | *Fatp4* | forward | ATGACTGCCTCCCCCTCTAC | 60 |
|  |  | reverse | AGTCATGCCGTGGAGTACG |  |
| Liver fatty acid binding protein  (L-FABP) | *Fabp1* | forward | ACTGGGGAAAAGGTCAAGGC | 60 |
|  |  | reverse | CCCAGTGTCATGGTATTGGTGAT |  |
| Intestinal fatty acid binding protein  (I-FABP) | *Fapb2* | forward | CCGAGAGATTTCTGGTAACGA | 60 |
|  |  | reverse | CAAGCTAGCCCTTCTGCATT |  |
| Inositol-requiring enzyme 1beta  (IRE-1β) | *Ire1b* | forward | TATTCCCGGCACAGAAGG | 60 |
|  |  | reverse | AGCCTGCAGAGAAGATATCCA |  |

| Target mRNA | Primer |  | Sequence (5'-3') | Annealing Temperature (℃) |
| --- | --- | --- | --- | --- |
| Ras-related GTPase 1b (SAR-1b) | *Sar1b* | forward | ATGGTCAGACAACAGGAAAGG | 60 |
|  |  | reverse | TGCACATGAACACTTCCAGAG |  |
| beta actin (β-actin) | *bactin* | forward | CTAAGGCCAACCGTGAAAAG | 60 |
|  |  | reverse | TACATGGCTGGGGTGTTGA |  |


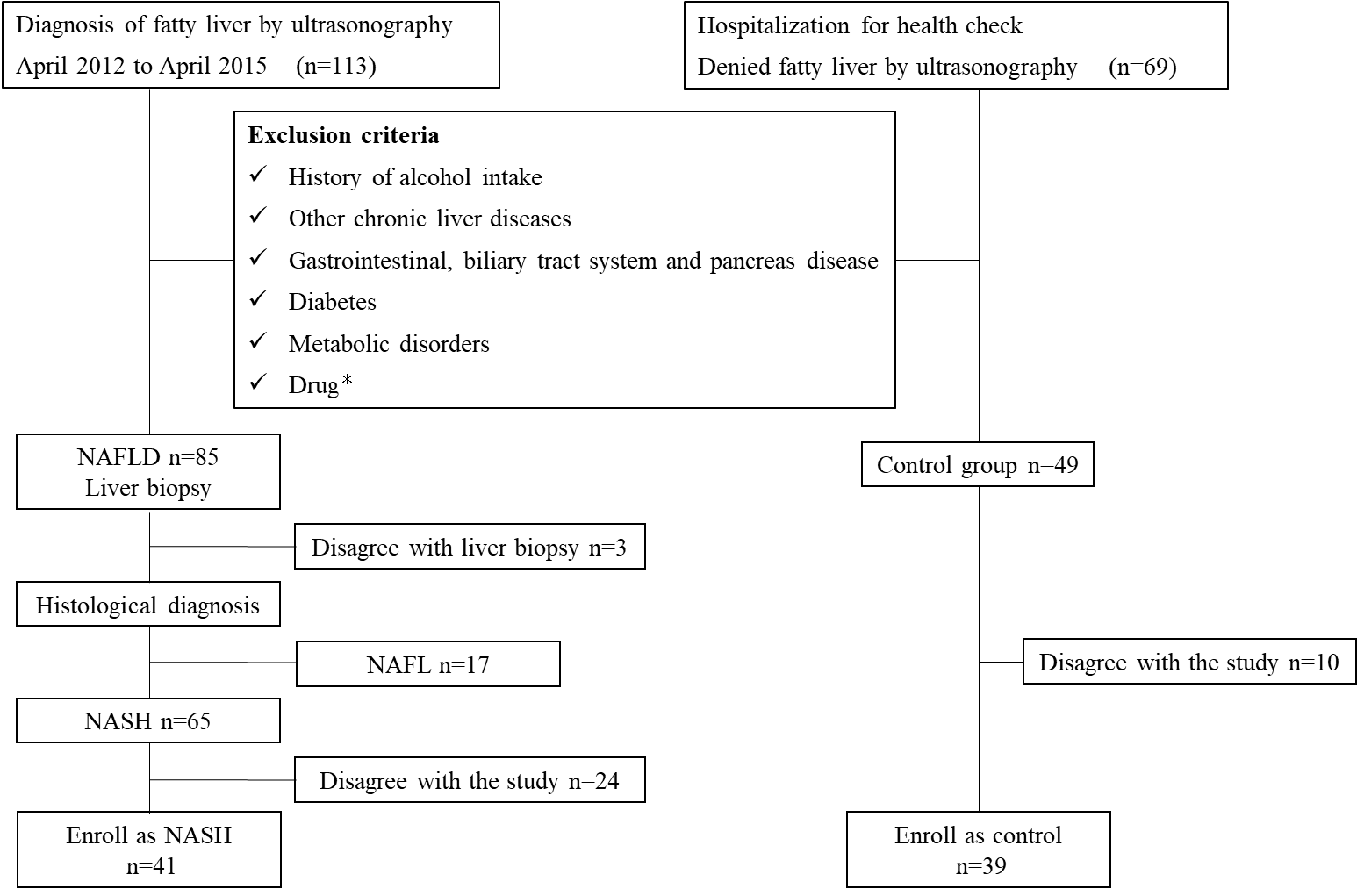


**Supplementary Figure S1.** Inclusion and Exclusion criteria

Subjects were excluded if their average daily alcohol intake was >20 grams for the 2 years. Additional exclusion criteria included any other chronic liver disease (e.g., positive hepatitis B surface antigen and positive antibody to hepatitis C virus), gastrointestinal disease, biliary tract system disease, acute or chronic pancreatic and renal disease, diabetes, metabolic disease, surgical or intrahepatic portal-systemic anastomosis, extrahepatic cholestasis, gastrointestinal surgery, systemic corticosteroids use, insulin use and drug use^*^. drug use^*^: b-blockers, glucocorticosteroids, insulin- lowering drugs, glucose- lowering drugs, lipid-lowering drugs, ursodeoxycholic acid, cholestyramine, other bile salt–chelating agents.


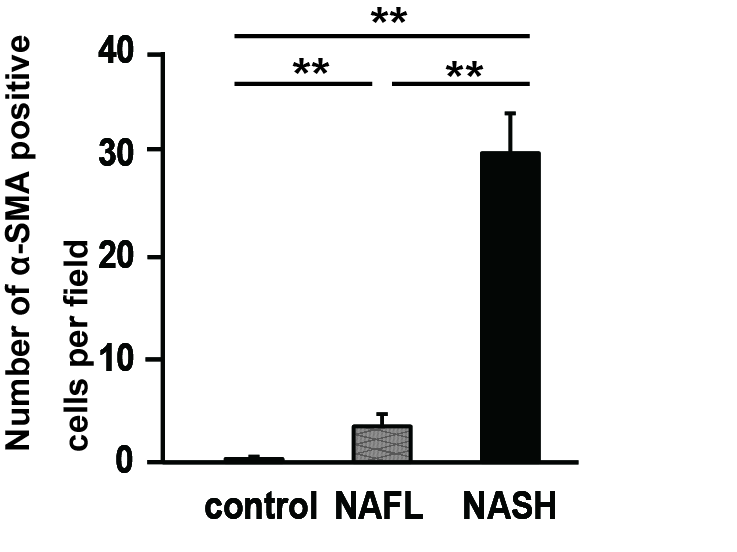


**Supplementary Figure S2.** Quantitative measurement of α-SMA positive cells (images from different five fields; N = 5 per group). **P < 0.01. Data in bar plots are expressed as the mean ± standard deviation. Statistical analysis between the three groups was performed using Kruskal-Wallis ANOVA. Only when a significant difference was found, Tukey's multiple comparison test was performed.


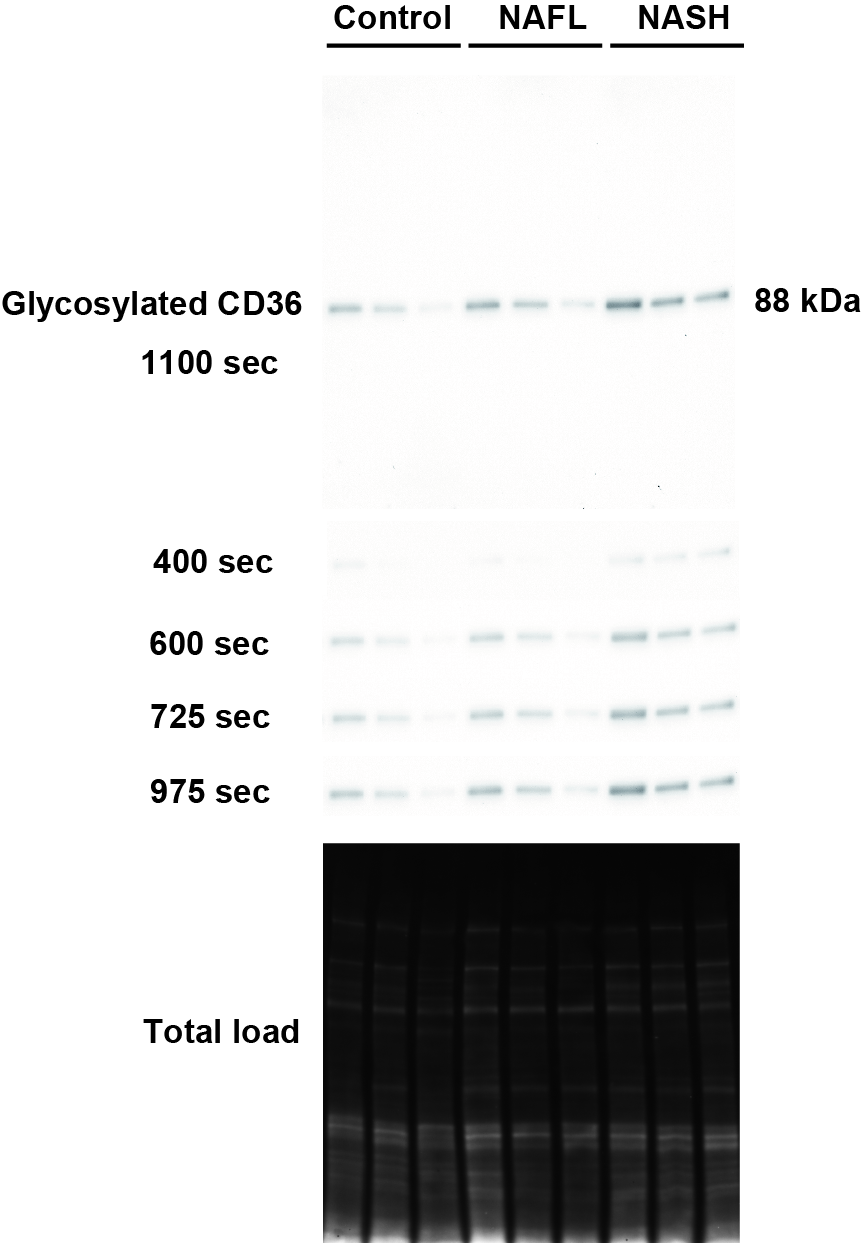


**Supplementary Figure S3.** Original blots for Figure 4B.

Blots with exposure times of 400 sec, 600 sec, 725 sec, 975 sec, and 1100 sec were described in parallel.


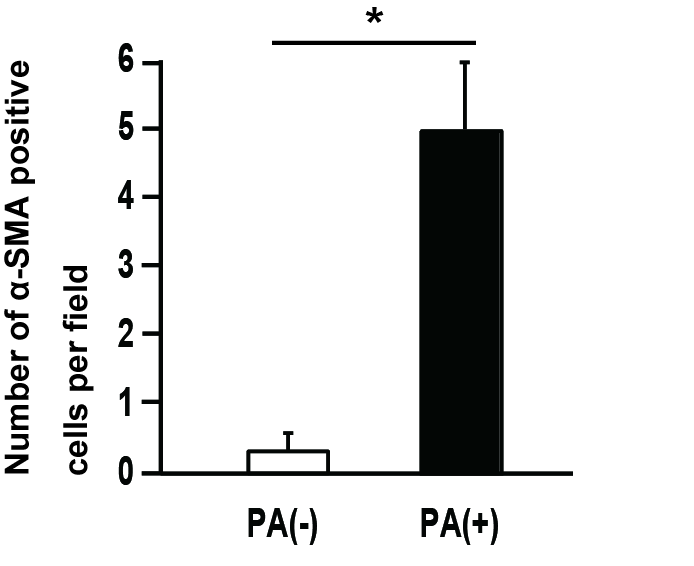


**Supplementary Figure S4**. Quantitative measurement of α-SMA positive cells (images from different five fields; N = 5 per group). *P < 0.05. Data in bar plots are expressed as the mean ± standard deviation. Significant differences were indicated by Mann-Whitney U-test. PA(+), PA solution group; PA(-), PA vehicle solution group.
